# Children of the State: The Nation's Debt to Its Warriors

**Published:** 1916-08-12

**Authors:** 


### CHILDREN OF THE STATE: THE NATION'S DEBT TO ITS WARRIORS.

It will be in the remembrance of our readers that we have continuously fought the battle of the wounded and disabled warriors of the nation, and that in the first year of the war, by a supreme effort, our plan to protect these warriors and their dependants from the stigma of charity and the disgrace of Poor-Law intermediaries, by constituting the Post Office the banker of these children of the State, was agreed to by the Government and ratified by the House of Commons. Under this arrangement the payment of the Government to the dependants of our warriors has for many months past been made through the Post Office, which has so become the banker of the wives and families of warriors at the Front, drawing their cheques every week and getting their allowances in advance. In this way the self-respect and thrift instincts of those immediately concerned have been safeguarded and encouraged to the great advantage of the nation, as well as to each dependant. Unfortunately, the position of the permanently disabled warriors of both services, and especially of old soldiers who may have been in receipt of pensions for services rendered in other wars, has been cruelly altered for the worse in several respects. The plan favoured by the Chancellor of the Exchequer, Mr. McKenna, would place the stigma of charity upon these noble men and their dependants, a course which must in practice prove an insult to our warriors and a disgrace to the nation and Empire by offering the former or their dependants charity and doles, while withholding payment by the State of the debt due to them, as forcibly set forth in a brochure entitled "The Nation: Its Warriors and Dependants," by Sir Henry Burdett, K.C.B., published by the Scientific Press, Limited, 28 and 29 Southampton Street, Strand, W.C. We have not space to set out all the facts, but copies of the brochure can be obtained from the publishers by those interested.

The position in a nutshell is this. As Parliament has bound employers in their private capacity, under the Employers' Liability Act, to provide for the injured workman, so it is essential that Parliament shall enforce upon the Government, as the employer in the most dangerous of all occupations, war, their liability to provide adequately, out of State funds, for all permanently disabled sailors and soldiers. It is a gross breach of trust and an insult to our brave warriors to offer them charity or doles in any form when disabled as a result of their courageous risking of their own lives in defence of the nation.

Instead of carrying out in practice Mr. Bonar Law's declaration at the Guildhall on September 4, 1914, that "it is essential that each of us should realise, as we have not always realised in the past, that our sailors and soldiers are children of the State, that they have the first claim upon the resources of our nation, and that it is the business of the State, and not of the individual, to treat its children properly and to provide for them adequately," the Chancellor of the Exchequer obstinately defends and upholds payment by the State of a flat rate pension, which is in many cases quite inadequate, without regard to the person's income or social status, supplemented by additional pensions, the money for which is to be obtained in large measure by voluntary contributions to be raised by appeals to the public; in other words, to maintain a system which will attach the stigma of charity to our warriors who may suffer or die in defence of the nation and Empire. We are glad that public opinion is utterly opposed to the Chancellor's proposals to introduce voluntary gifts to diminish the State's monetary liability to our disabled sailors and soldiers. Mr. W. W. Rutherford, M.P., properly criticises the present regulations of the Government pensions as unfair and pettifogging. Speaking as the representative of the chief municipalities and county councils, he declared that they would resist to the uttermost any attempt to use local, private, trade union, or benevolent funds in order to reduce the State pensions and allowances. The State must do its duty first, and then there would still remain many deficiencies to be supplied by local and private funds.

Mr. McKenna claims "fully to share the sentiments in regard to the duty of the State towards those who had shed their blood in our service. He had never had but one object at the back of everything else, and that was to get this question settled during the war, while the public and the House of Commons were generous." Why is it, then, that Mr. McKenna retires behind the Naval and Military War Pensions Act, 1915, which was keenly contested and strongly objected to at the time of its passing? Further, he must be aware that the experience gained in the working of this Act demonstrates its incompleteness and many injustices, together with the urgent necessity there is for the amendment of the Royal Warrant, which, in fact, should be withdrawn and an intelligible, human code substituted for it.

These two sentences embody the general feeling in the House of Commons on Tuesday last, and they are likely to find expression and insistence, as we hope, in the Press throughout the country. The National Committee and representatives of the chief municipalities and county councils will have their opportunity when they confer with the Statutory Committee and the Government in the course of the ensuing week. If the best interests of our fighting men of both services, their wives and dependants, are to be adequately safeguarded and the wishes of the people throughout the nation and Empire are to prevail, the Government, through the Chancellor of the Exchequer, will promptly eliminate the element of voluntary contributions from the proposals for additional pensions to the flat rate. In this way alone can the duty of the State, to look after these disabled sailors and soldiers of all ranks, and to care for their dependants while the men are serving or when they are killed, be adequately discharged.

